# Violence Against Healthcare Workers: A Worldwide Phenomenon With Serious Consequences

**DOI:** 10.3389/fpubh.2020.570459

**Published:** 2020-09-18

**Authors:** Sandro Vento, Francesca Cainelli, Alfredo Vallone

**Affiliations:** ^1^Faculty of Medicine, University of Puthisastra, Phnom Penh, Cambodia; ^2^Raffles Medical Group Clinic, Phnom Penh, Cambodia; ^3^Infectious Diseases Unit, G. Jazzolino Hospital, Vibo Valentia, Italy

**Keywords:** violence, healthcare worker (HCW), doctor-patient relationship, nurse-patient relationship, workplace

## Introduction

Verbal and physical violence against healthcare workers (HCWs) have reached considerable levels worldwide, and the World Medical Association has most recently defined violence against health personnel “an international emergency that undermines the very foundations of health systems and impacts critically on patient's health” (1). Two systematic reviews and meta-analyses published at the end of 2019 found a high prevalence of workplace violence by patients and visitors against nurses and physicians (2), and show that occupational violence against HCWs in dental healthcare centers is not uncommon (3).

## Recent Studies

In the first study (2), the authors systematically searched PubMed, Embase, and Web of Science from their inception to October 2018, and included 253 eligible studies (with a total of 331,544 participants). 61.9% of the participants reported exposure to any form of workplace violence, 42.5% reported exposure to non-physical violence, and 24.4% experienced physical violence in the past year. Verbal abuse (57.6%) was the most common form of non-physical violence, followed by threats (33.2%) and sexual harassment (12.4%). The prevalence of violence against HCWs was particularly high in Asian and North American countries, in Psychiatric and Emergency departments, and among nurses and physicians (2).

In the second study (3), a systematic review and analysis of the literature was done using PubMed, ScienceDirect, Scopus, Web of Science, Cochrane Library and ProQuest. Original articles published between January 1992 and August 2019 and written in English were included in the analysis. The violence experienced by dental healthcare workers was both physical and non-physical (shouting, bullying, and threatening) and also included sexual harassment (3), and in most cases, male patients, or coworkers were responsible. Violent events ranged from 15.0 to 54.0% with a mean prevalence of 32%, and physical abuse ranged from 4.6 to 22% (3).

Most recently, the World Medical Association has condemned the increasingly reported cases of health care workers being attacked because of the fear that they will spread SARS-CoV-2. The situation in India is particularly shocking, with health care workers stigmatized, ostracized, discriminated against, and physically attacked, but incidents have been reported across the world, for instance from France, Mexico, Philippines, Turkey, UK, Australia, and USA (4, 5).

## Discussion

The recent systematic reviews and meta-analyses and the World Health Organization condemnation of the attacks against HCWs treating patients with COVID-19 have confirmed the seriousness of the situation regarding violence against doctors and nurses worldwide. Many countries have reported cases of violence, and some are particularly affected by this problem. A Chinese Hospital Association survey collecting data from 316 hospitals revealed that 96% of the hospitals surveyed experienced workplace violence in 2012 (6), and a study done by the Chinese Medical Doctor Association in 2014 showed that over 70% of physicians ever experienced verbal abuse or physical injuries at work (7). An examination of all legal cases on violence against health professionals and facilities from the criminal ligation records 2010–2016, released by the Supreme Court of China, found that beating, pushing, verbal abuse, threatening, blocking hospital gates, and doors, smashing hospital property were frequently reported types of violence (8). In India, violence against healthcare workers and damage to healthcare facilities has become a debated issue at various levels (9), and the government has made violence against HCWs an offense punishable by up to 7 years imprisonment, after various episodes of violence and harassment of HCWs involved in COVID-19 care or contact tracing (10). In Germany, severe aggression or violence has been experienced by 23% of primary care physicians (11). In Spain, there has been an increase in the magnitude of the phenomenon in recent years (12). In the UK, a Health Service Journal and UNISON research found that 181 NHS Trusts in England reported 56,435 physical assaults on staff in 2016–2017 (13). In the USA, 70–74% of workplace assaults occur in healthcare settings (14). In Italy, in just one year, 50% of nurses were verbally assaulted in the workplace, 11% experienced physical violence, 4% were threatened with a weapon (15); 50% of physicians were verbally, and 4% physically, assaulted (16). In Poland, Czech Republic, Slovakia, Turkey many nurses have been physically attacked or verbally abused in the workplace (17). According to the South African Medical Association, over 30 hospitals across South Africa reported serious security incidents in just 5 months in 2019 (18), and in Cape Town violence against ambulance crews is widespread (19). In Iran, the prevalence of physical or verbal workplace violence against emergency medical services personnel is 36 and 73% respectively (20). The World Health Organization lists Australia, Brazil, Bulgaria, Lebanon, Mozambique, Portugal, Thailand as other countries where studies on violence directed at HCWs have been conducted (21).

The consequences of violence against HCWs can be very serious: deaths or life-threatening injuries (15), reduced work interest, job dissatisfaction, decreased retention, more leave days, impaired work functioning (22), depression, post-traumatic stress disorder (23), decline of ethical values, increased practice of defensive medicine (24). Workplace violence is associated directly with higher incidence of burnout, lower patient safety, and more adverse events (25).

Which are the most at-risk services and what are the underlying factors of this growing violence? Emergency Departments, Mental Health Units, Drug and Alcohol Clinics, Ambulance services and remote Health Posts with insufficient security and a single HCW are at higher risk. Working in remote health care areas, understaffing, emotional or mental stress of patients or visitors, insufficient security, and lack of preventative measures have been identified as underlying factors of violence against physicians in a 2019 systematic review and meta-analysis (26).

In public hospital/services, insufficient time devoted to patients and therefore insufficient communication between HCWs and patients, long waiting times, and overcrowding in waiting areas (27), lack of trust in HCWs or in the healthcare system, dissatisfaction with treatment or care provided (26), degree of staff professionalism, unacceptable comments of staff members, and unrealistic expectations of patients and families over treatment success (28) are thought to contribute. Indeed, in public hospitals worldwide, staff shortages prevent front-line HCWs from adequately coping with patients' demands. In private hospitals/services, too extended hospital stays, unexpectedly high bills, prescription of expensive and unnecessary investigations are key factors. Finally, the media frequently report extreme cases of possible malpractice and portray them as representative of “normal” practice in hospitals (24).

What can be done to reduce the escalating violence against HCWs? HCWs worldwide generally advocate for more severe laws, but harsher penalties alone are unlikely to solve the problem. Importantly, evidence on the efficacy of interventions to prevent aggression against doctors is lacking, and a systematic review and meta-analysis found that only few studies have provided such evidence (29). Just one randomized controlled trial indicated that a violence prevention program decreased the risks of patient-to-worker violence and of related injury in hospitals (30), whereas contrasting results in violence rates after implementation of workplace violence prevention programs have been observed from longitudinal studies (29). There is no evidence on the effectiveness of good place design and work policies aimed to reduce long waiting times or crowding in waiting areas (29). More studies are clearly needed to provide evidence-based recommendations, and interdisciplinary research with the involvement of anthropologists, sociologists, and psychologists should be encouraged. However, certain measures have to be taken and can be corrected, should they be shown as ineffective in properly conducted studies.

Security measures have been advocated for years (31) and should be taken to safeguard particularly the most at-risk services. First, staff shortages, so common in public hospitals worldwide, should be acted upon, and increased funding should be allocated to employ more doctors and nurses. Hence, the duration of each patient encounter would be augmented, particularly in overburdened public hospitals, allowing the (often young) (32) doctors to develop a meaningful relationship with the patient. Second, healthcare organizations and universities should considerably improve the communication skills of current and future HCWs to reduce unrealistic expectations or misunderstanding of patients and families. Third, HCWs who denounce any verbal or physical violence should be fully supported by their healthcare organizations; this would reduce the huge issue of under-reporting of workplace violence (33, 34). Good courses should be organized for HCWs to learn how to identify early signs that somebody may become violent, how to manage dangerous situations, and how to protect themselves.

Prompt communication about delays in service provision should be given to patients and their relatives when waiting times are long because certain conditions are prioritized. Alarms and closed-circuit televisions should be placed in the higher-risk departments and in areas where doctors and/or nurses work in isolation. Sanctioning of violence by patients, relatives or visitors must be imposed. Staff should be increased and security officers should be placed, particularly at night, in remote Health Posts and Emergency Departments and at particular times (violence tends to happen in the evenings/nights, when more patients under the influence of drugs and alcohol present); the number of night shifts should be limited (23). Efforts should be made to improve job satisfaction of HCWs (25). Finally, media should cease to contribute to the general public's distrust toward HCWs and institutions. Many patients report their negative experiences of medical care to news or media outlets which are highly interested in these stories and very often do not check the information before publishing it (24). These biased media reports may exacerbate the tension.

All workers have a right to be safe on their job, and healthcare workers are no exception. The idea that violence is inherent to doctors and nurses' work, especially in certain departments, needs to be fought; urgent measures must be implemented to ensure the safety of all HCWs in their environment, and the needed resources must be allocated. Failure to do so will worsen the care that they are employed to deliver and will ultimately negatively affect the whole healthcare system worldwide.

## Author Contributions

SV had the idea of writing the manuscript and drafted it. FC co-drafted the manuscript. AV contributed to the drafting, and reviewed the manuscript. All the authors approved the final version.

## Conflict of Interest

The authors declare that the research was conducted in the absence of any commercial or financial relationships that could be construed as a potential conflict of interest.
